# Signatures of positive selection in the *cis*-regulatory sequences of the human oxytocin receptor (*OXTR*) and arginine vasopressin receptor 1a (*AVPR1A*) genes

**DOI:** 10.1186/s12862-015-0372-7

**Published:** 2015-05-13

**Authors:** Helmut Schaschl, Susanne Huber, Katrin Schaefer, Sonja Windhager, Bernard Wallner, Martin Fieder

**Affiliations:** Department of Anthropology, University of Vienna, Vienna, Austria; Cognitive Science Platform at the University of Vienna, Vienna, Austria; Department of Behavioural Biology, University of Vienna, Vienna, Austria

**Keywords:** Oxytocin receptor, Arginine vasopressin 1a receptor, Positive selection, Balancing selection, Genetic diversity

## Abstract

**Background:**

The evolutionary highly conserved neurohypophyseal hormones oxytocin and arginine vasopressin play key roles in regulating social cognition and behaviours. The effects of these two peptides are meditated by their specific receptors, which are encoded by the oxytocin receptor (*OXTR*) and arginine vasopressin receptor 1a genes (*AVPR1A*), respectively. In several species, polymorphisms in these genes have been linked to various behavioural traits. Little, however, is known about whether positive selection acts on sequence variants in genes influencing variation in human behaviours.

**Results:**

We identified, in both neuroreceptor genes, signatures of balancing selection in the *cis*-regulative acting sequences such as transcription factor binding and enhancer sequences, as well as in a transcriptional repressor sequence motif. Additionally, in the intron 3 of the *OXTR* gene, the SNP rs59190448 appears to be under positive directional selection. For rs59190448, only one phenotypical association is known so far, but it is in high LD’ (>0.8) with loci of known association; i.e.*,* variants associated with key pro-social behaviours and mental disorders in humans.

**Conclusions:**

Only for one SNP on the OXTR gene (rs59190448) was a sign of positive directional selection detected with all three methods of selection detection. For rs59190448, however, only one phenotypical association is known, but rs59190448 is in high LD’ (>0.8), with variants associated with important pro-social behaviours and mental disorders in humans. We also detected various signatures of balancing selection on both neuroreceptor genes.

**Electronic supplementary material:**

The online version of this article (doi:10.1186/s12862-015-0372-7) contains supplementary material, which is available to authorized users.

## Background

Human behaviours are characterised by marked differences on both the population and individual level. The two evolutionary-conserved neurohypophyseal hormones oxytocin and arginine vasopressin and their receptors play key roles in modulating cognition and social behaviours in mammals [1]. The corresponding genes are expressed mainly in the hypothalamus and secreted from the posterior pituitary gland. The main hormonal activity of oxytocin is to regulate parturition and lactation. Vasopressin, in contrast, regulates salt and water homeostasis. The neurophysiological effects of oxytocin are shown in facilitating social communication, affiliative behaviours, and social cognition. Accordingly, oxytocin has been reported to influence maternal behaviour, trust, generosity, empathy, eye contact, face memory, and also to reduce anxiety [2]. Vasopressin expressed in the brain modulates social behaviours in sexually dimorphic ways, including anxiety-related behaviour as shown in animal studies, and may act to enhance social recognition and the emotional response to stress [3–5]. Similar effects have also been reported in humans, especially with regard to aggression and the perception of anger [6]. For instance, cerebrospinal fluid levels of vasopressin are directly correlated with general aggression and aggression against persons [7]. Vasopressin may influence aggression in males by priming individuals to respond to emotionally ambiguous social stimuli as if they were threatening or aggressive [8]. Additionally, genetic variation within the *AVPR1A* gene, particular in the 5′ upstream region (5′ UTR) in both humans and chimpanzees, has been linked to relationship quality and pair bonding [9], personality traits [1,10,11], and autism disorders [12,13]. In particular, polymorphic microsatellites in the 5′ flanking promoter region have been associated with species-specific and individual variation in social behavior in mice [14]. Furthermore, two SNPs (rs7294536 and rs10877969) have been identified in the promoter region of the human *AVPR1A* gene, which were significantly associated with autism in Korean subjects [13].

The *physiological effects* of these two neuroactive peptides are mediated via their specific receptor molecules, encoded by the oxytocin receptor gene (*OXTR*) and arginine vasopressin receptor 1a gene (*AVPR1A*). These two genes share a feature unique to the G protein–coupled receptor genes: an intron located before the seventh transmembrane domain of the receptor encoding sequences [1]. In several species, *polymorphisms* in these genes, mainly in the non-coding regions, have been linked to inter-individual variation in cognition and social behaviours [1,15].

Recently, it has been reported that polymorphisms in the OXTR gene are associated with the development of psychopathy, for example in children with conduct problems or with callous and unemotional traits [16]. Intronic SNPs on OXTR are further associated with autistic spectrum disorder (ASD). Nonetheless. alleles appear to have opposite effects in Asian compared to Caucasian populations [17–19]. Polymorphisms in and near the 3′UTR is also involved in ASD risk [9,20,21]. A recent meta-analysis including 3941 individuals from 11 independent samples and 16 SNPs on OXTR, however, found a significant association with ASD for only 4 SNPs (i.e., rs7632287, rs237887, rs2268491, rs2254298) [22]. In addition, specific sequence variants of OXTR are associated with the Asperger Syndrome [23]. Finally, an allelic variation of rs53576 on OXTR apparently influences human empathy [24], and various SNP variations are associated with maternal sensitivity [25] and parental touch provision [26]; rs7632287 on OXTR plays also a role in pair-bonding [27].

Additionally, support seeking is associated with rs53576 on OXTR as well, but again different in Caucasian Americans compared with Koreans [28]. Rs53576 is also associated with pro-sociality: Kogan et al. [29] reported that individuals homozygous for the G allele on rs53576 were rated more pro-social than carriers of the A allele. Moreover, rs53576 influences trust behaviour [30], stress reactivity [24], and lower self- reported affect [31]. There is also evidence that OXTR SNPs (rs2228485, rs53576) help process social information from the emotion expression of faces and the voice [24,31].

In economic games, SNPs on OXTR play a role, too: Israel et al. [32] demonstrated that the SNPs rs1042778, rs2268490, and rs237887 on OXTR intron 3 are associated with performance in the dictator game and the social value task. Although this association also holds true in an independent female sample [32], in a Swedish population (performing the dictator and the trust-game) neither associations reported by Isreal et al. [32], nor any association with rs53576, could be confirmed. Extensive reviews of OXTR associations can be found in [1] and [33].

Thus, oxytocin and its specific receptor are crucial for a range of social behaviours and disorders associated with social dis-functioning. Moreover, oxytocin-related cultural differences in social behaviour exist among different human populations [28].

In summary, these studies suggest that genetic variation, particularly in the non-coding DNA, of both neuroreceptor genes significantly influences social behaviour in humans. Little, however, is known about whether positive selection may have shaped sequence variation at loci involved in social cognition. In most studies, genes that show signatures of *positive selection* usually include loci involved in host–pathogen interactions, reproduction, dietary adaptation, and skin and hair coloration [34].

Only two studies have so far reported positive selection for genes involved in human behaviours. Ding et al. [35] suggested that the seven-repeat variant of the dopamine receptor gene D4 (DRD4 7R allele) originated as a rare mutational event, but increased to high frequency in human populations due to positive selection (but see [36]). The allele DRD4 7R is reportedly associated with certain psychiatric phenotypes such as the attention-deficit hyperactivity disorder (ADHD) [37,38]. The other study reported an excess of high frequency-derived haplotypes for the monoamine oxidase A gene (MAO-A), perhaps reflecting recent positive selection [39]. This gene shows association with borderline mental retardations, depression, and psychopathy [40–42].

In the present study, we thus examined whether positive selection has shaped the polymorphism in the *OXTR* and *AVPR1A* genes*.* We utilized large SNP datasets released by the 1000 Genomes project (http://www.1000genomes.org/) [43]. We used the degree of genetic differentiation (measured by *F*_*ST*_) over many worldwide populations as a proxy for positive selection at the SNP level. This approach enables detecting the signature of recent positive selection in human populations. We further investigated whether any SNPs identified under selection are known to be associated with human behavioural traits.

## Results

### Regression trees

**Fig. 1:**
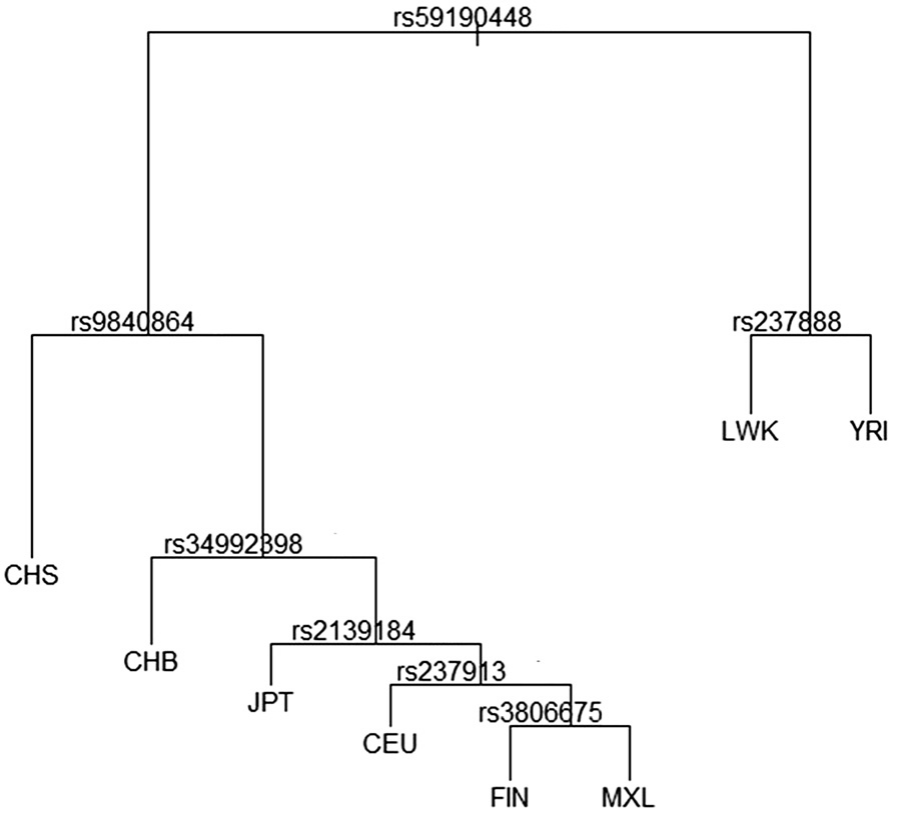
Regressions tree of the 122 SNPs on OXTR regressing on the 14 human populations from 1000genomes. The regression trees shows the SNPs (nodes) according to which the 14 population do separate from each other

The OXTR gene sample of the 14 analysed human populations from the 1000 Genomes project is separated by nine SNPs, dividing the tree consecutively (rs59190448, rs11706648, rs9840864, rs34992398, rs7610471, rs2139184, rs237916, rs35498753, rs237902). Starting with rs59190448, the tree unfolds from node to node (SNPs), dividing the populations as shown in Fig. 1. In the *AVPR1a* gene, the seven SNPS (rs3759292, rs7980289, rs34310119, rs11174816, rs11174817, 11174818, rs182223193) unfold the regression tree (Fig. 4).

### Detecting selection in the OXTR gene

**Fig. 2:**
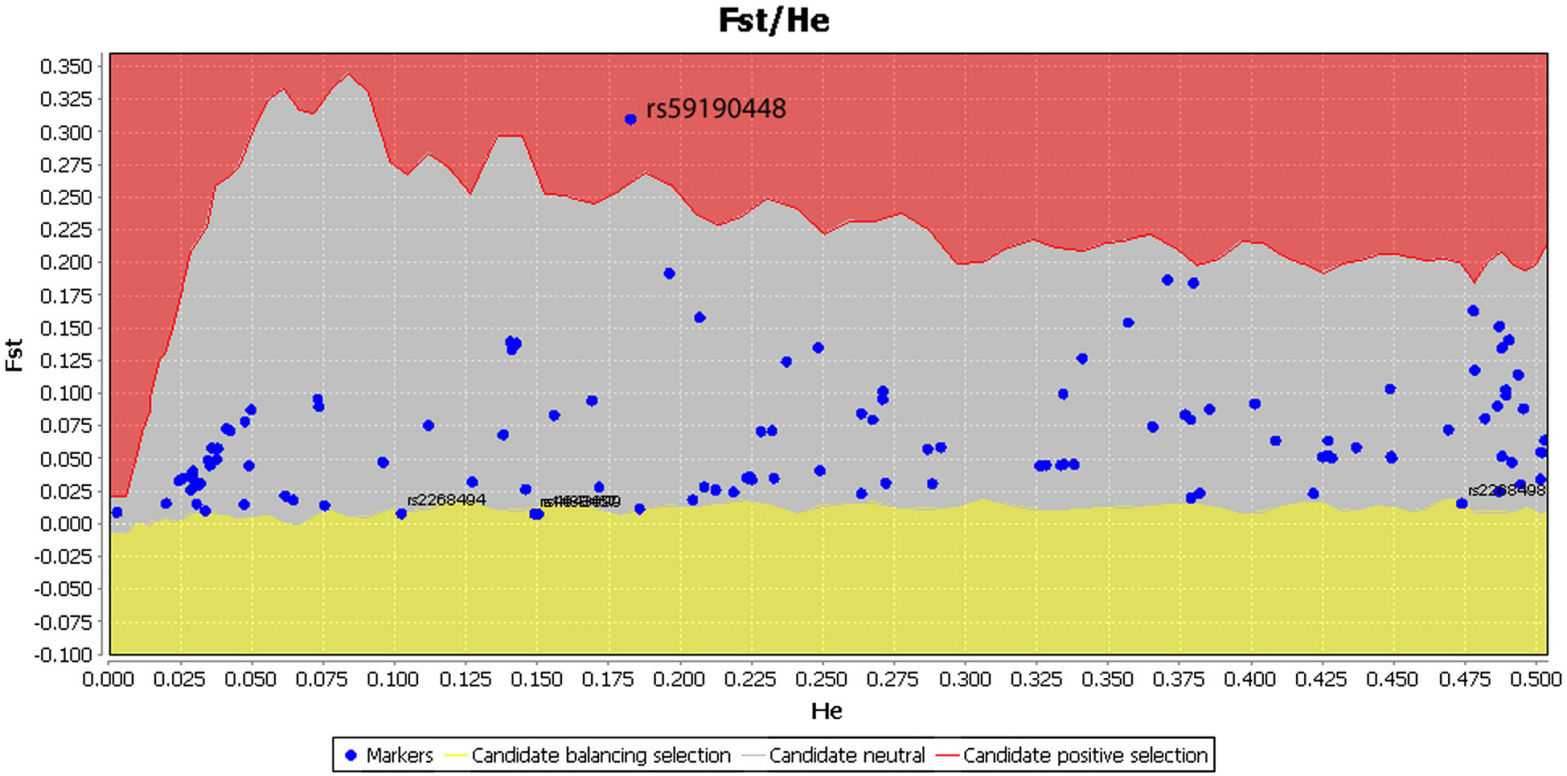
Detection of outlier SNPs of the OXTR gene using FDIST. FDIST suggests the SNPs rs2268494, rs2268498, rs4643699, and rs1488467 are under balancing selection, whereas SNP rs59190448 is under positive directional selection. x-axis: estimated heterozygosity values; y-axis: FST-values. The red area indicates positive selection, the grey area neutrality, and the yellow area balancing selection. Confidence intervals represent borders between “selection areas”

**Fig. 3:**
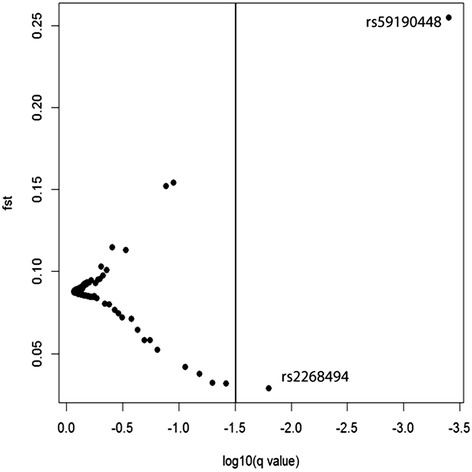
Detection of outlier SNPs in the OXTR gene using BayeScan. BayeScan indicates ‘very strong’ evidence for selection (the vertical line corresponds to log10(BF) > 1.5) for the two loci rs59190448 and rs2268494. Log 10(q value) are shown on the x-axis and *FST*-values on the y axis. *FST*: locus-specific genetic divergence among human populations. Log10(q-value): decision factor in logarithmic scale (base 10) to determine selection

All three approaches (FDIST, BayeScan and the extended Lewontin and Krakauer test (FLK)) identified the SNP rs59190448 (in the intron 3) to be significantly under positive directional selection (Table 1). Furthermore, FDIST and BayeScan detected the SNP rs2268494 in the *OXTR* gene under balancing selection (Table 1, Fig. 2 and Fig. 3, Additional file 1: Table S1).

**Table 1:** Identified outlier loci in the OXTR gene that are under balancing selection and directionaldirectionalselection using the approaches FDIST, BayeScan and F.LK

		**FDIST**			**BayeScan**				**FLK**	
**Locus**	**Function**	***H***_***E***_	***F***_***ST***_	***P***	**log10(BF)**	***q***	**α**_**i**_	***F***_***ST***_	***F.LK***	***F.LK.p.val***
rs2268494	Intron 3	0.1022	0.0083	0.0000	1.8492	0.0159	−1.3815	0.0291		
rs59190448	Intron 3	0.1825	0.3103	0.0004	3.3977	0.0004	1.447	0.2548	13.521	0.001
rs2268498	Upstream	0.4736	0.0159	0.0009						
rs4643699	Upstream	0.15	0.0081	0.0000						
rs1488467	Upstream	0.1487	0.0081	0.0000						

The results with significance *P* value < 0.005 are shown for FDIST and F.LK; * the BayeScan log10(BF) > 1.5 indicating ‘very strong’ evidence (*P*(α ≠ lo = 0.03 → 0.01) for selection. The SNP rs59190448 is under directionalselection and the remaining SNPs under balancing selection

The SNP rs59190448 also unfolds the regression tree (Fig. 1) and is characterised by an *F*_*ST*_ *>* 0.25. In the populations with African origin (populations ASW, LWK and YRI), rs59190448 appears as the homozygote AA allele (ancestral allele). The heterozygote allele AG is mostly present in African populations and only occasionally in two European populations (CEU, GBR). In all the other populations, rs59190448 is present only as the homozygote GG allele. Interestingly, the Neanderthal genome sequence (Assembly and Analysis tracks on UCSC Genome Browser) is present as the homozygote GG allele at this locus, whereas the Denisova genome sequence appears as the homozygote AA allele. From the 1000 Genomes data, we further calculated that rs59190448 is in high LD (>0.8) with an array of SNPs of known social behavioural associations, namely rs237889, rs13316193, rs2254298, and rs1042778.

The SNP rs2268494, which is under balancing selection, is located within a CTCF (zinc finger protein) transcription factor binding sequence. The CTCF protein acts as a transcriptional regulator and is implicated in genomic imprinting. In addition, FDIST detected the upstream gene SNP variants rs4643699 and rs1488467 to be under balancing selection (Table 1, Fig. 2).

### Detecting selection in the AVPR1A gene

**Fig. 4:**
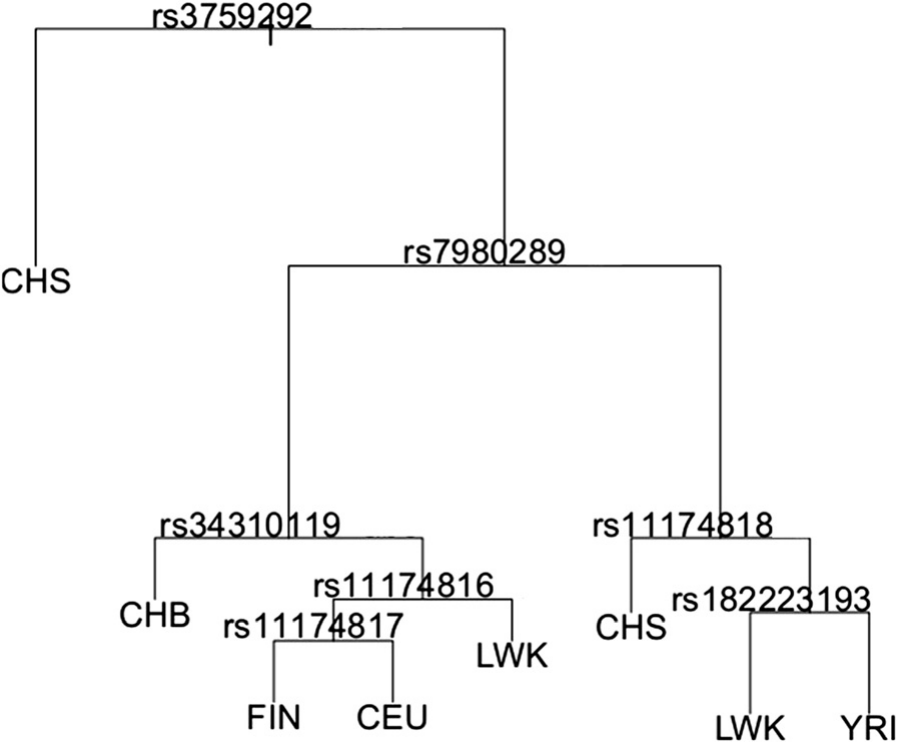
Regressions tree of the 86 SNPs of the AVPR1a regressing on the 14 human populations from 1000genomes. The regression trees shows the SNPs (nodes) according to which the 14 population do separate from each other

Even though some SNPs on the *AVPR1A* gene unfold the regression tree (Fig. 4), no SNPs were detected under directional selection by the FDIST and BayeScan approaches. The FLK test, however, detected the SNP rs3759292, which is located in the promotor region of AVPR1A, as being under directional selection (Table 2).

**Table 2:** Identified outlier loci in the AVPR1A gene that are under balancing selection and directional selection using the approaches FDIST, BayeScan and F.LK

		**FDIST**			**BayeScan**				**FLK**	
**Locus**	**Function**	***H***_***E***_	***F***_***ST***_	***P***	**log10(BF)**	***q***	**α**_**i**_	***F***_***ST***_	***F.LK***	***F.LK.p.val***
rs12830859	Downstream	0.2631	0.0143	0.0000	1.1382	0.0455	−1.2413	0.0271		
rs1587098	Downstream	0.1412	0.0111	0.0000						
rs1580705	Downstream	0.1311	0.0176	0.0008						
rs11174805	Downstream	0.1412	0.0111	0.0000						
rs11174806	Downstream	0.1412	0.0111	0.0000						
rs11174807	Downstream	0.1431	0.0124	0.0000						
rs117072097	3′UTR	0.0272	0.0041	0.0013						
rs11174811	3′UTR	0.1467	0.0047	0.0000	1.2742	0.0336	−1.3077	0.0255		
rs34462214	Intron	0.1549	0.0048	0.0000	1.5306*	0.0252	−1.3346	0.0248		
rs3021529	5′UTR	0.1567	0.0057	0.0001	1.1260	0.0270	−1.2497	0.0270		
rs3759292	Promoter								14.1	0.001
rs7308855	Upstream	0.1114	0.0020	0.0000	2.1158*	0.0076	−1.6966	0.0178		

The results with significance *P* value < 0.005 are shown for FDIST and F.LK; * the BayeScan log10(BF) > 1.5 indicating ‘very strong’ evidence (*P*(α ≠ .5 = 0.03 → 0.01) for selection. All SNPs except SNP rs3759292 are under balancing selection

**Fig. 5:**
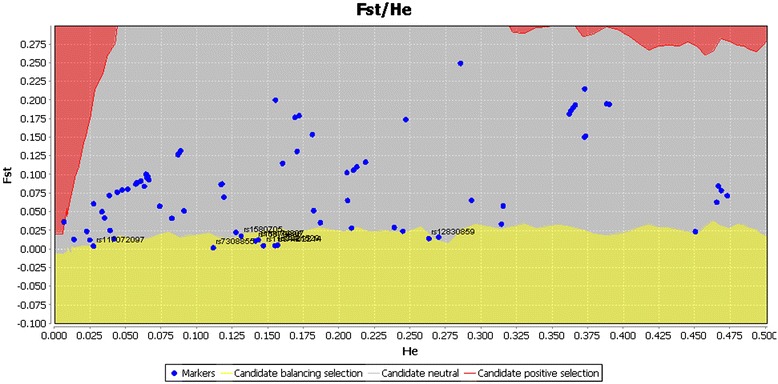
Detection of outlier SNPs of the AVPR1A gene using FDIST. FDIST suggests the SNPs rs12830859, rs1587098, rs1580705, rs11174805, rs11174806, rs11174807, rs117072097, rs11174811, rs34462214, rs3021529, and rs7308855 are under balancing selection. x-axis: estimated heterozygosity values; y-axis: *F*_*ST*_-values. The red area indicates positive selection; grey area neutrality, and yellow area balancing selection. Confidence intervals represent borders between “selection areas”

**Fig. 6:**
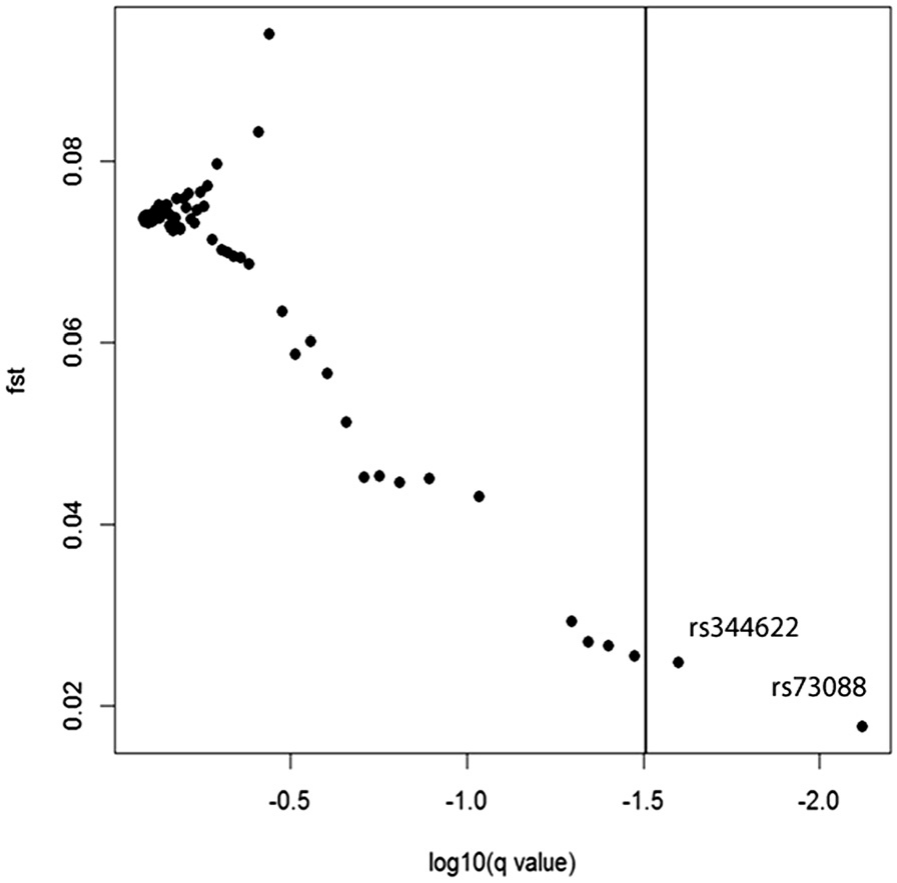
Detection of outlier SNPs in the AVPR1A gene using BayeScan. BayeScan indicates ‘very strong’ evidence for selection (the vertical line corresponds to log10(BF) > 1.5) for the two loci rs344622 and rs7308855. Log 10(q value) are shown on the x-axis, *F*_*ST*_-values on the y axis. *F*_*ST*_: locus-specific genetic divergence among human populations. Log10(q-value): decision factor in logarithmic scale (base 10) to determine selection

This result should be treated with caution because only FLK detected this SNP as being under positive selection. Nonetheless, FDIST and BayScan identified the SNP rs34462214 (in the intron) and the SNP rs7308855 (upstream variant) to be under balancing selection (Table 2, Fig. 4, Fig. 5, Fig. 6). The APVR1A upstream SNP rs7308855 is located within the transposable element hAT-Charlie family DNA transposon (MER103C), which is an element found only in mammals and plays an important role in gene regulation [33]. Furthermore, FDIST also identified several other SNPs as being significantly (*P* < 0.005) under balancing selection. The BayeScan approach detected at the level of log 10(BF) 1 → 1.5 (i.e., ‘strong evidence’ for selection), congruent with FDIST, the following SNPs as being under selection: rs12830859, rs11174811, and rs3021529 (Table 2). In Additional file 1: Table S1 and Additional file 1: Table S2, *F*_*ST*_ values and detailed results from FDIST and BayeScan for all loci on OXTR, respectively on AVPR1 are displayed.

## Discussion

This study shows that, the *OXTR* intronic SNP rs2268494 detected as being under balancing selection is associated with reactions to betrayal [44] as well as the autism spectrum disorder (ASD [21]). Interestingly, this SNP is located within a CTCF (zinc finger protein) transcription factor binding motif, which is recognised by the CTCF (CCCTC-binding factor (zinc finger protein)) transcription factor. This transcriptional regulator protein, which can activate or repress transcription, is associated with diseases that include mental retardation [45]. It remains unknown whether this transcription factor influences the gene expression of the *OXTR* gene.

In the *AVPR1A* gene, at least two of the three approaches detected the SNPs rs34462214 and rs7308855 as being under balancing selection. For the SNP rs34462214, no phenotypic associations are reported yet. The SNP rs7308855, however, is located in a DNA transposon sequence region (a hAT-Charlie family DNA transposon) found only in placental mammals [46]. This transposon family a) has the epigenetic signatures of enhancers, insulators, and repressors, b) directly binds transcription factors essential for pregnancy, and c) coordinately regulates gene expression in response to progesterone and cAMP [46]. The latter study concluded that this transposable element contributed to the origin of a novel gene regulatory network dedicated to pregnancy in placental mammals.

On AVPR1a the signature of balancing selection could be adaptive because of the pleiotropic function of this gene. In addition, FDIST (as well as BayeScan with log 10(BF) 1 → 1.5, i.e., ‘strong evidence’ for selection) detected congruently several more SNPs under selection in the *AVPR1A* gene. From these SNPs under selection, rs11174811 and rs3021529 are located in important regulatory sequences. The SNP rs11174811 is located in potential microRNA binding sites [35]. Studies suggest that microRNA binding sites in the 3′UTR are potent regulators of gene expression in animals. Indeed, this SNP has been found to be associated with expression levels of the *AVPR1A* gene in human brain tissue [47]. Interestingly, this locus is associated with risk of drug use disorders in humans [47]. The SNP rs3021529 is located within several transcription factor binding sequences, such as for the enhancer and for the transcriptional repressor that repress neuronal genes in non-neuronal tissues. It remains unknown whether this SNP influences the expression of this gene. Nonetheless, as we had access to SNP data only from 1000 Genomes, we were unable to include the repeat regions in the promotor of AVPR1a, previously identified as being related to social behaviour, in our analysis. This prohibits drawing definitive conclusions about a particularly directional selection of AVPR1a (e. g., because we think that the SNP detected as being under positive selection should be treated with caution).

Besides balancing selection, we also found strong evidence of positive directional selection. This was detected by all three used approaches in the intron 3 of the OXTR gene (SNP rs59190448), indicating adaptive evolution at this locus. Very recently, Myers et al. [48] found evidence that rs59190448 modulates the relationship between early life stress and the risk of anxiety later in life. Additionally, rs59190448 is in high LD (>0.8) with an array of SNPs of known social behavioural associations including: mind-reading, empathy ([49,50], rs13316193), psychopathy ([16], rs1042778, rs2254298), autism spectrum disorder ([45], rs237889; [9], rs2254298), ADHD ([51], rs13316193), ([52], rs2254298) and pro-social behaviour in cooperation games ([32], rs1042778).

Interestingly a comparison among primate species reveals that, in humans, the reference allele of rs59190448 is G, but A in the chimp, orang-utan and the macaque. Among these latter species, rs59190448seems to be in an area of accelerated evolution ([53] - http://genome-euro.ucsc.edu/cgi-bin/hgTracks?db=hg19&position=chr3%3A8802751-8803251&hgsid=207867630_3B0oNUMNn7N4rYbLDRmof2xHeoqR).

The regression tree of the genotype regressing on the ethnical affiliation for the *OXTR* gene may hint at actual population differentiation. The SNP rs59190448 splits the regression tree at its root into the African populations LWK and YRI on one branch and all the other populations on the other branches. The branch with the other non-African populations is consecutively split, separating the two Chinese populations (CHS, CHB) from the others, by rs9840864*,* recently found to be associated with reactions to betrayal [44]. This is followed by the Japanese population (JPT), then the European populations (CEU, FIN) and the Mexican population (MXL) – suggesting a further differentiation into populations (Fig. 1) according to allele frequencies. The question now is whether the branching is reflected in population differences in behaviour. Although the direct link between behaviour and variation in the *OXTR* gene is still missing, one hint might come from population differences in human socio-spatial behaviour: interpersonal proximity and touch varies between so-called contact cultures [e.g., Arabs, Latin Americans and Southern Europeans – proximity in descending order] and non-contact cultures [e.g., Asians and Northern Europeans – proximity in descending order] (see [54] for a review).

Although we found no signs of positive selection for the intronic SNP rs53576, we did find that it is in LD’ at 0.9696 with rs59190448 in Europeans, and in LD’ at 0.959 with rs2324728 and rs237884 in East Asians. And indeed, rs53576 on OXTR has been identified as having a different allelic association depending on the cultural context. This was already exemplified in the introduction section: according to [28], the emotionally sensitive G allele carriers seek more social support than the AA genotypes, but only in European Americans. In East Asians, in contrast, where emotional support seeking is not the cultural norm, this behaviour is not related to rs53576. Kim et al. [28] further found that different alleles of rs53576 are differently associated with emotional suppression in European Americans and Koreans: among Koreans, emotional suppression was associated with the rs53576 GG genotype, which is characterized as more socio-emotionally sensitive, but not with the AA genotype. In contrast, Americans with the GG genotype were less engaged in emotional suppression compared to those with the AA genotype. Ethnic differences between East Asians and Europeans have also been found for an autistic spectrum disorders risk allele on OXTR (rs2254298; reviewed in [1] and [18]). Finally, oxytocin influences the inter-individual distance between men and women. In an experiment including only female participants, oxytocin-treated women significantly reduced the “comfortable distance” to a male experimenter but not to a female experimenter [55]. Based on this result, it could be speculated – and tested by future studies – that the differences on the *OXTR* gene also contribute to the known cultural differences in body contact and individual space [56,57]. Nonetheless, these cultural differences do not preclude that second-generation immigrants adopt local norms if they are well-integrated into their host societies.

Additionally, differences in OXTR alleles could result in different regulation of social behaviours and emotions among diverse human cultures. For example, facial expressions are important cues used in social interactions and interpretations of emotional states [58]. Interestingly, the perception of facial signals is not universal among human societies [59]. Jack et al. [60] found differences in the organization of the conceptual space of emotions between East Asians and Western Caucasians. Interestingly, a very recent study showed, for OXTR, a relationship to emotional empathy, whereas AVPR1a was associated with cognitive empathy [61]. Accordingly, our results suggest that molecular biological differences in OXTR alleles may also be related to different perception or cognition of emotions among different socio-ecologically adapted societies. We may therefore speculate that directional selection processes on OXTR are also associated with the ability to perceive different emotional states (e.g., expressed as facial signals) in diverse human societies. The cognitive part of emotional perception mediated via AVPR1a, however, could be universal for all human societies. As noted above, conclusions on selection on AVPR1a might be limited because the repetitive sequences in the promotor region were not included in our analysis.

Cultural differences are also found in ASD, which is associated with a preference for greater inter-individual distance [62]. ASD has a lower prevalence in Afro-American children than in children with Caucasian ancestry [63,64]. Nonetheless, any conclusions that ethnic differences in ASD are associated with selection on the OXTR gene would be premature: despite several SNPs on OXTR having been identified as being associated with ASD (reviewed in [22]), the association of OXTR with ASD is weaker than the association of ASD with other genes. The Simons Research Foundation for Autism Research (http://sfari.org/) identified 284 human genes associated with ASD. Compared with other genes involved in ASD, OXTR scores only with “suggestive evidence” (scoring data and scoring methods explained at: https://gene.sfari.org/autdb/GS_Statistics.do). At least in African populations, however, rs59190448 is in high LD’ (>0.8) with rs2254298, the SNP, for which an association with autism has been primarily described by Wu et al. [17] and confirmed in a meta-analysis by [22].

Of special interest are our results of the positive directional selection of one SNP located in intron 3 and the balancing selection in *cis*-regulative sequences in the 1receptor. These selection processes might reflect adaptations to diverse climate environments or climate changes per se. Darwin noted the challenges of climate changes for the behaviour and survivorship in human evolution in relation to natural selection and compared theses challenges with other animals [65]. Such climate changes have a tremendous influence on terrestrial habitats and therefore on resource availability and body physiology [66,67]. While major genetic changes in body metabolism could have lethal results, behavioural changes for better adaptation to survive in different environments seem to be less risky. Our results could reflect such adaptation processes in humans living under different environmental conditions, i.e.*,* signatures of balancing selection stabilize metabolic processes, and directional selection processes enable these populations to develop adaptive behavioural strategies. Both processes would improve survivorship and reproductive success.

We used very stringent criteria to test for positive selection in the present study to minimise false positives. We might therefore actually underestimate the level of positive selection acting on regulative sequences in both genes. Indeed, a recent study suggests that *balancing selection*, as a natural consequence of frequent adaptation, is a crucial force maintaining genetic variation [68]. Many functionally important sequence variants are located in this *cis*-regulatory sequences, playing an important role in the evolution of species-specific expression of genes, and very likely also in individual-specific variation within species. Transcription factor binding sites and other *cis*-regulatory sequences can be particularly important in the evolutionary adaptation of humans. This is because regulatory elements apparently contribute substantially to both adaptive substitutions and deleterious polymorphisms, with key implications for human evolution and disease [69]. Adaptation-driven balanced polymorphisms could therefore be an important source of consequential genetic variation at genes involved in social cognition.

Balancing selection could be particularly beneficial in fast-changing environments in which diverse human social behaviour patterns might provide an evolutionary adaptive advantage. However, certain variants under selection are linked to detrimental behaviours in humans. These genes with their pleotropic function may therefore maintain balanced polymorphisms that become deleterious in a certain environmental context. The identification of specific positive selection forces on SNPs relevant to gene expression and social behaviour is a promising novel direction for formulating hypotheses about driving forces and constraints in the evolution of human behaviour and social bonding. Likewise, this new approach can provide explanations and context-dependent predictions about within- and between-culture variation in non-verbal behaviour such as interpersonal proximity.

To date, research has strongly concentrated on identifying the genetic basis of social cognition, behaviour, and mental diseases on an individual basis. We assume that the evidence that particularly positive selection has been acting at a SNP on the OXTR gene highlights the importance of a “population perspective”. Here, differences between populations due to evolutionary processes should be kept in mind when investigating the genetic predisposition of human behaviour, cognition, and mental diseases. A hint about such differences could be the culturally dependent effects of rs53576 and rs2254298 on emotional support seeking, empathy, and ASD [22,28], both in high LD’ with rs59190448. This call for taking potential selection pressures during the relatively recent evolution of *Homo sapiens* into account.

More generally, the detection of selection in human genes relevant to behaviour could be a starting point for investigating phenotypical associations. This would involve searching for behavioural and cognitive associations with SNPs detected as being under selection as well as SNPs being in high LD’ with a SNP under selection.

## Conclusions

This study investigates – based on 1000 Genomes data and using three established methods of selection detection – if a positive selection pressure has been acting on the oxytocin receptor gene (OXTR) and vasopressin receptor gene (AVPR1a). Only for one SNP on the OXTR gene (rs59190448) was a sign of positive directional selection detected with all three methods of selection detection. For rs59190448, however, only one phenotypical association is known, but rs59190448 is in high LD’ (>0.8), with variants associated with important pro-social behaviours and mental disorders in humans. We also detected various signatures of balancing selection on both neuroreceptor genes. Possible causes for these findings during the evolution and migration of *Homo sapiens* are discussed, ranging from climate adaptations to social behaviour.

## Methods

### Genetic dataset

The UCSC Genome Browser database/data (http://genome.ucsc.edu/) were used to retrieve genomic data. The *OXTR* gene*,* located on chromosome 3 (p25.3), spans approximately 19 kilobases (kb) and comprises four exons and three introns. Exons 1 and 2 correspond to the 5′ untranslated region (5′ UTR), followed by exons 3 and 4 encoding the 389 amino acids of the receptor; intron 3, which is the largest at 12 kb, separates the coding region immediately after the sixth transmembrane-spanning domain, encoded by exon 3. Exon 4 encodes the seventh transmembrane domain, the C terminus, and the entire 3′ untranslated region (3′ UTR) [70]. The *AVPR1A* gene is located on chromosome 12 (q14.2) and spans approximately 10 kb, comprising 2 exons; both exons encode the 418 amino acids of the receptor. In this gene, a single intron is located between the exons which encode the sixth and the seventh transmembrane domains [71]. We included single nucleotide polymorphism (SNP) data derived from the 1000 Genomes database (http://www.1000genomes.org/) of 14 human populations from Africa, Asia, Europe, and America (for population abbreviations and studied samples per population see Additional file 1: Table S3), with a minor allele frequency (MAF) of > 0.05 from the human populations listed in Additional file 1: Table S3, totalling 122 SNPs and 86 SNPS across the entire *OXTR* gene and *AVPR1A* gene, respectively. We included about 5 kb upstream (including promoter regions) and 2 kb downstream sequences of the coding sequences (genomic position in accordance to the reference genome GRCh37/hg19 on UCSC Genome Browser: *OXTR:* chr3:8,787,095-8,813,300; *AVPR1A*: chr12:63,531,539-63,548,590). We retrieved the SNP data from the 1000 Genome database using Engine SPSmart v5.1.1 (http://spsmart.cesga.es/engines.php?dataSet=engines) [72]. Each genotype was encoded as diploid nucleotides. For all following analyses, we used the population abbreviations according to 1000 Genomes (see Additional file 1: Table S3). We decided to include a small number of base pairs upstream and downstream of the coding region of the genes in order to include only *cis*- regulative acting sequences such as promoters and enhancer sequences.

### Genetic statistical analyses and tests for selection

We performed the following analyses:

A) To identify the SNPs separating the 14 analysed populations, we calculated a regressing tree of the SNPs regressing on the 14 population identifiers using the R library r-part (R version 3.0.1) [73].

B) We used two different *F*_*ST*_-based approaches to detect loci with a pattern deviating from neutrality. Loci that are under balancing selection are expected to show unusually low levels of genetic differentiation (i.e., *F*_*ST*_ < 0.05), while loci under directional selection are expected to show unusually high levels of differentiation (i.e., *F*_*ST*_ > 0.25) among populations. Based on the analysed 14 human populations (*n* = 1092) from the 1000 Genomes database, we aimed to identify the SNPs under selection by employing the two different *F*_*ST*_-based methods: FDIST [74] and BayeScan [75]. Firstly, FDIST calculations were done in the programme LOSITAN (http://popgen.net/soft/lositan/) [76]. LOSITAN evaluates the relationship between *F*_*ST*_ and expected heterozygosity (*H*_*E*_), describing the expected distribution of Wright’s inbreeding coefficient *F*_*ST*_ vs. *H*_*E*_ under an island model of migration with neutral markers; this distribution is used to identify outlier loci that have excessively high or low *F*_*ST*_ compared to neutral expectations. In this study, we simulated the neutral distribution of *F*_*ST*_ with 100,000 iterations at a significance *P* value < 0.005 (*P* (Simulation *F*_*ST*_ < sample *F*_*ST*_); runs were performed using the infinite allele model. LOSITAN also implements a multiple testing correction based on false discovery rates (FDR) to avoid overestimating the percentage of outlier loci. Secondly, we used the programme BayeScan 2.1 (http://cmpg.unibe.ch/software/bayescan/), which uses a Bayesian statistics approach, to detect outlier loci under selection [75]. This approach assumes that genetic differentiation among populations in different environments is different for loci under selection than for the remaining genome. The programme estimated the posterior probability of a given locus being under the effect of selection by defining two alternative models, one that includes the effect of selection and another that excludes it. Selection is introduced by decomposing locus–population *F*_*ST*_ coefficients into a population-specific component (*β*_*i*_) shared by all loci, and a locus-specific component (*α*_*i*_) shared by all the populations, using a logistic regression. Significantly positive values of *α*_*i*_ suggest directional selection, and significantly negative values suggest balancing selection (or purifying selection) (for details see [75]). The respective posterior probabilities of these two models are estimated using a reversible-jump Markov Chain Monte Carlo (MCMC) approach. The posterior probability that a locus is subjected to selection (P (*α*_*i*_ ≠ 0)) is then estimated from the MCMC output by counting the number of times *α*_*i*_ is included in the model. Each Markov chain was run for 100,000 updating steps, after 20 pilot runs of 10,000 iterations each and an initial burn-in of 1,000,000 steps. Samples were collected every 50 steps (thinning). The programme defines a q-value, which is the FDR analogue of the *P* value. In the present study we chose as the criterion a log10 Bayes Factor (BF) as the FDR Jeffreys’ scale of evidence for Bayes factors = “very strong”which corresponds to a range of q-values of 0.03-0.01, providing very strong evidence that a locus is under selection (programme manual: http://cmpg.unibe.ch/software/bayescan/). The BayeScan programme provides an R script which was used to control for the FDR and to generate the graphical outputs of the BayeScan results. Additionally, because the error rate of the *F*_*ST*_ outlier detection in Lositan and BayeScan has been criticized recently [77], we included an additional approach to verify the SNPs detected as being under positive selection by FDIST and BayeScan, the “extended Lewontin and Krakauer test” (FLK). This test compares patterns of differences between allele frequencies in several populations to their expectation under neutral evolution. The null hypothesis of neutral evolution assumes a tree structure with branch length corresponding to the amount of genetic drift in each population (F). The tree is estimated from the matrix of Reynold’s genetic distances between populations, using the neighbour joining (NJ) algorithm (for details see [78]); the approach is implemented in the FLK programme, which can be found at https://qgsp.jouy.inra.fr/index.php?option=com_content&view=article&id=50&Itemid=55). As FLK uses the calculated allele frequencies, we included the allele frequencies provided by Engine SPSmart v5.1.1 based on the “1000genome continents” (AFRICA, EUROPE, ASIA and AMERICA) only; therefore, the number of SNPs MAF > 0.05 included in FLK differs from the number of SNPs included in Lositan and BayeScan (Additional file 1: Table S4 and Additional file 1: Table S5).

C) We further investigated whether any SNPs identified as being under selection are known to influence a phenotypic trait, or whether SNPs known to influence phenotypic traits are in linkage disequilibrium (LD) with the SNPs identified as being under selection (LD threshold > 0.8).

## Additional file

Additional file 1:
**Includes the following supplementary tables:** **Table S1.** FST values, FDIST and BayeScan results for each tested loci of the OXTR gene. **Table S2.** FST values, FDIST and BayeScan results for each tested loci of the AVPR1a gene. **Table S3.** Population and sample size used in the study. **Table S4.** Results of the Lewontin and Krakauer test extended for the SNPs on the OXTR gene. **Table S5.** Results of the Lewontin and Krakauer test extended for the SNPs on the AVPR1a gene.

## Abbreviations

*OXTR*: Oxytocin receptor
*AVPR1A*: Arginine vasopressin receptor 1a
SNP: Single nucleotide polymorphism
LD: Linkage disequilibrium
5′ UTR: 5′ upstream region
DRD4 7R allele: Seven-repeat variant of the dopamine receptor gene D4
ADHD: Attention-deficit hyperactivity disorder
MAO-A: Monoamine oxidase A gene
F_ST_: Fixation index
kb: kilobases
3′ UTR: 3′ untranslated region
MAF: Minor allele frequency
HE: Expected heterozygosity
FDR: False discovery rate
MCMC: Markov Chain Monte Carlo
BF: Bayes Factor
FLK: Extended Lewontin and Krakauer test
NJ: Neighbour joining
