# Supplementary material for: Signatures of positive selection in the cis-regulatory sequences of the human oxytocin receptor (OXTR) and arginine vasopressin receptor 1a (AVPR1A) genes
Source: BMC Evol Biol. 2015 May 13;15:85. doi: 10.1186/s12862-015-0372-7 (PMC4429470; doi:10.1186/s12862-015-0372-7)
Supplement: Additional file 1: — Includes the following supplementary tables: Table S1. FST values, FDIST and BayeScan results for each tested loci of the OXTR gene. Table S2. FST values, FDIST and BayeScan results for each tested loci of the AVPR1a gene. Table S3. Population and sample size used in the study. Table S4. Results of the Lewontin and Krakauer test extended for the SNPs on the OXTR gene. Table S5. Results of the Lewontin and Krakauer test extended for the SNPs on the AVPR1a gene. [file 12862_2015_372_MOESM1_ESM.docx]

**Supplementary data**

**Table S1.**  FDIST and BayeScan results for each tested loci of the OXTR gene. Left part shows expected heterozygosity (*H_E_*) and *F_ST_* values per loci for any significant (*P* (Simulation *F_ST_* < sample *F_ST_*) outlier loci under diversifying selection and balancing selection detected by FDIST. Right part of the table shows the α-factors and *F_ST_* values per loci detected by BayeScan. SNPs identified in BayeScan as under balancing (α_i_ < 0) and/or diversifying selection (α_i_ > 0) with the corresponding posterior estimate of *F_ST_* are shown as well as q-values; a Bayes Factor (BF) above 32 (i.e., log10(BF) > 1.5) corresponds to a posterior probability of > 0.97 🡪 0.99 and thus indicates ‘very strong’ evidence for selection. Note: *F_ST_* values differ due to different calculation algorithms.

| **Locus** | *H_E_* | ***F_ST_*** | ***P*** | **log10(BF)** | **q-val** | **α_i_** | ***F_ST_*** |
| --- | --- | --- | --- | --- | --- | --- | --- |
| rs59746083 | 0.2480 | 0.1353 | 0.0973 | -0.8510 | 0.5193 | 0.0796 | 0.0952 |
| rs56898713 | 0.2371 | 0.1245 | 0.1575 | -0.9625 | 0.5472 | 0.0559 | 0.0930 |
| rs35014760 | 0.1714 | 0.0285 | 0.9552 | -1.2066 | 0.7791 | -0.0220 | 0.0865 |
| rs2324728 | 0.3705 | 0.1871 | 0.0066 | 0.4612 | 0.1115 | 0.6700 | 0.1544 |
| rs4493422 | 0.3407 | 0.1271 | 0.1305 | -0.7361 | 0.4715 | 0.1055 | 0.0977 |
| rs1126908 | 0.0496 | 0.0878 | 0.2889 | -1.0133 | 0.6212 | 0.0536 | 0.0933 |
| rs9872310 | 0.2281 | 0.0712 | 0.5545 | -1.3463 | 0.8385 | 0.0048 | 0.0882 |
| rs9872425 | 0.1456 | 0.0269 | 0.9502 | -1.1865 | 0.7639 | -0.0214 | 0.0865 |
| rs11131147 | 0.1271 | 0.0325 | 0.8834 | -1.3079 | 0.8194 | -0.0096 | 0.0872 |
| rs237884 | 0.3796 | 0.1851 | 0.0067 | 0.4461 | 0.1305 | 0.6522 | 0.1524 |
| rs6770632 | 0.3567 | 0.1545 | 0.0449 | -0.3203 | 0.2942 | 0.2607 | 0.1131 |
| rs1042778 | 0.4776 | 0.1635 | 0.0149 | -1.2907 | 0.8133 | 0.0161 | 0.0891 |
| rs36047964 | 0.1557 | 0.0838 | 0.3959 | -1.3177 | 0.8250 | 0.0076 | 0.0884 |
| rs2139184 | 0.1689 | 0.0949 | 0.3246 | -1.2512 | 0.7997 | 0.0152 | 0.0891 |
| rs237885 | 0.5013 | 0.0555 | 0.7288 | 0.1385 | 0.1806 | -0.6004 | 0.0581 |
| rs237886 | 0.5026 | 0.0644 | 0.6218 | -0.4847 | 0.3693 | -0.2081 | 0.0768 |
| rs11706648 | 0.4281 | 0.0508 | 0.812 | -1.2292 | 0.7920 | -0.0193 | 0.0866 |
| rs11718289 | 0.4484 | 0.1038 | 0.2444 | -1.2993 | 0.8179 | 0.0123 | 0.0888 |
| rs237887 | 0.4875 | 0.0522 | 0.805 | -1.0478 | 0.6771 | -0.0421 | 0.0854 |
| rs2268490 | 0.3852 | 0.0886 | 0.39 | -1.3370 | 0.8302 | -0.0081 | 0.0872 |
| rs237888 | 0.1960 | 0.1925 | 0.0202 | -0.6614 | 0.4348 | 0.1349 | 0.1009 |
| rs147229047 | 0.0351 | 0.0453 | 0.5662 | -1.2503 | 0.7978 | 0.0096 | 0.0888 |
| rs62243363 | 0.0304 | 0.0155 | 0.9125 | -1.2004 | 0.7692 | 0.0022 | 0.0883 |
| rs918316 | 0.2320 | 0.0717 | 0.5498 | -1.2235 | 0.7900 | -0.0186 | 0.0866 |
| rs9840864 | 0.4867 | 0.1516 | 0.0351 | -1.3916 | 0.8488 | 0.0068 | 0.0883 |
| rs4686301 | 0.4083 | 0.0641 | 0.6494 | -1.3570 | 0.8417 | -0.0045 | 0.0875 |
| rs62243364 | 0.0304 | 0.0155 | 0.9125 | -1.2098 | 0.7857 | 0.0014 | 0.0883 |
| rs114435510 | 0.0310 | 0.0296 | 0.669 | -1.0496 | 0.6827 | 0.0394 | 0.0920 |
| rs75975944 | 0.0642 | 0.0185 | 0.9764 | -1.2309 | 0.7940 | -0.0124 | 0.0871 |
| rs115787100 | 0.0318 | 0.0310 | 0.6491 | -1.0236 | 0.6519 | 0.0506 | 0.0933 |
| rs9810278 | 0.0408 | 0.0735 | 0.3557 | -1.0364 | 0.6587 | 0.0526 | 0.0934 |
| rs2268491 | 0.3378 | 0.0460 | 0.8458 | -1.3257 | 0.8263 | -0.0074 | 0.0873 |
| rs2268492 | 0.3767 | 0.0840 | 0.4478 | -1.3308 | 0.8289 | 0.0061 | 0.0883 |
| rs62243365 | 0.0334 | 0.0104 | 0.9879 | -0.9719 | 0.5599 | -0.0549 | 0.0850 |
| rs62243366 | 0.0334 | 0.0104 | 0.9879 | -0.9734 | 0.5718 | -0.0623 | 0.0846 |
| rs2300549 | 0.4942 | 0.0307 | 0.9571 | -1.0204 | 0.6449 | -0.0517 | 0.0850 |
| rs2268493 | 0.3786 | 0.0802 | 0.4735 | -1.3668 | 0.8438 | 0.0015 | 0.0879 |
| rs116571980 | 0.0615 | 0.0220 | 0.9263 | -1.2477 | 0.7959 | -0.0049 | 0.0876 |
| rs78062775 | 0.0642 | 0.0184 | 0.9769 | -1.1486 | 0.7461 | -0.0273 | 0.0862 |
| rs2268494 | 0.1022 | 0.0083 | 1 | 1.8492 | 0.0159 | -1.3815 | 0.0291 |
| rs2254298 | 0.3331 | 0.0455 | 0.8514 | -1.2590 | 0.8033 | -0.0105 | 0.0871 |
| rs2254295 | 0.3341 | 0.0460 | 0.8463 | -1.3177 | 0.8250 | -0.0104 | 0.0871 |
| rs60902022 | 0.4860 | 0.0907 | 0.3677 | -1.3380 | 0.8314 | -0.0057 | 0.0874 |
| rs139574523 | 0.0198 | 0.0159 | 0.7932 | -1.1050 | 0.7165 | 0.0137 | 0.0897 |
| rs57329700 | 0.2066 | 0.1584 | 0.0633 | -1.3411 | 0.8327 | 0.0110 | 0.0887 |
| rs237889 | 0.4268 | 0.0641 | 0.6584 | -1.3790 | 0.8458 | -0.0022 | 0.0877 |
| rs60345038 | 0.4817 | 0.0813 | 0.4671 | -1.2697 | 0.8051 | -0.0147 | 0.0868 |
| rs78172575 | 0.0753 | 0.0145 | 0.9944 | -0.9040 | 0.5335 | -0.0754 | 0.0839 |
| rs58102519 | 0.0733 | 0.0902 | 0.3199 | -1.1926 | 0.7666 | 0.0236 | 0.0900 |
| rs13316193 | 0.4890 | 0.0988 | 0.2979 | -1.3267 | 0.8276 | -0.0032 | 0.0876 |
| rs11131148 | 0.4890 | 0.0988 | 0.2979 | -1.3559 | 0.8406 | -0.0045 | 0.0875 |
| rs62243369 | 0.3279 | 0.0455 | 0.8239 | -1.2842 | 0.8085 | -0.0121 | 0.0870 |
| rs62243370 | 0.3279 | 0.0455 | 0.8239 | -1.2861 | 0.8101 | -0.0125 | 0.0870 |
| rs11131149 | 0.4889 | 0.1027 | 0.262 | -1.3506 | 0.8396 | -0.0023 | 0.0876 |
| rs114641034 | 0.0374 | 0.0498 | 0.5255 | -1.1108 | 0.7206 | 0.0335 | 0.0912 |
| rs59190448 | 0.1825 | 0.3103 | 0.0004 | 3.3977 | 0.0004 | 1.4470 | 0.2548 |
| rs113264292 | 0.0283 | 0.0263 | 0.72 | -0.5446 | 0.3920 | 0.2446 | 0.1151 |
| rs13319411 | 0.3259 | 0.0448 | 0.8307 | -1.2581 | 0.8015 | -0.0148 | 0.0869 |
| rs58933435 | 0.0728 | 0.0963 | 0.2862 | -1.2043 | 0.7767 | 0.0221 | 0.0899 |
| rs237890 | 0.4910 | 0.0476 | 0.8483 | -1.4417 | 0.8535 | -0.0001 | 0.0878 |
| rs12631502 | 0.3340 | 0.1000 | 0.2996 | -1.4166 | 0.8516 | -0.0037 | 0.0875 |
| rs34992398 | 0.2708 | 0.1021 | 0.2671 | -1.3453 | 0.8373 | 0.0018 | 0.0879 |
| rs237891 | 0.4688 | 0.0724 | 0.5708 | -1.2805 | 0.8068 | -0.0108 | 0.0871 |
| rs78519233 | 0.0357 | 0.0587 | 0.4566 | -1.0525 | 0.6881 | 0.0466 | 0.0927 |
| rs13326924 | 0.0487 | 0.0449 | 0.5985 | -1.0193 | 0.6374 | 0.0552 | 0.0934 |
| rs77943865 | 0.0471 | 0.0152 | 0.9607 | -1.0438 | 0.6713 | -0.0461 | 0.0853 |
| rs53576 | 0.4781 | 0.1181 | 0.145 | -1.3089 | 0.8222 | -0.0100 | 0.0871 |
| rs57793364 | 0.0257 | 0.0353 | 0.6019 | -1.1362 | 0.7394 | -0.0048 | 0.0880 |
| rs237892 | 0.2911 | 0.0591 | 0.6858 | -1.3847 | 0.8478 | -0.0071 | 0.0873 |
| rs35498753 | 0.2489 | 0.0412 | 0.8842 | -1.1037 | 0.7122 | -0.0348 | 0.0857 |
| rs151463 | 0.4488 | 0.0520 | 0.7991 | -1.2074 | 0.7814 | -0.0214 | 0.0865 |
| rs7652281 | 0.2865 | 0.0578 | 0.7006 | -1.1472 | 0.7428 | 0.0306 | 0.0905 |
| rs185922247 | 0.0026 | 0.0092 | 0.5 | -0.8026 | 0.4886 | 0.1255 | 0.1030 |
| rs237893 | 0.4491 | 0.0507 | 0.8138 | -1.1522 | 0.7493 | -0.0240 | 0.0863 |
| rs11711703 | 0.2881 | 0.0312 | 0.9468 | -1.2082 | 0.7836 | -0.0211 | 0.0865 |
| rs113202287 | 0.0280 | 0.0370 | 0.5856 | -1.1543 | 0.7523 | 0.0168 | 0.0898 |
| rs73132848 | 0.1403 | 0.1398 | 0.1042 | -1.2898 | 0.8118 | 0.0124 | 0.0888 |
| rs237894 | 0.2673 | 0.0802 | 0.4477 | -1.3790 | 0.8458 | 0.0038 | 0.0881 |
| rs237895 | 0.4901 | 0.1411 | 0.0523 | -1.4366 | 0.8525 | 0.0033 | 0.0880 |
| rs2268495 | 0.3787 | 0.0202 | 0.9939 | 0.2853 | 0.1539 | -0.7412 | 0.0526 |
| rs78828026 | 0.0957 | 0.0477 | 0.6763 | -1.2194 | 0.7879 | -0.0147 | 0.0869 |
| rs61183828 | 0.1423 | 0.1386 | 0.1069 | -1.2993 | 0.8179 | 0.0120 | 0.0888 |
| rs76548613 | 0.0378 | 0.0581 | 0.4609 | -1.0696 | 0.6933 | 0.0415 | 0.0921 |
| rs6767512 | 0.2326 | 0.0353 | 0.9174 | -1.0750 | 0.6983 | -0.0392 | 0.0856 |
| rs2268496 | 0.3818 | 0.0237 | 0.988 | -0.0753 | 0.2310 | -0.4701 | 0.0646 |
| rs237897 | 0.4876 | 0.1350 | 0.0727 | -1.4010 | 0.8507 | 0.0016 | 0.0879 |
| rs79896191 | 0.1409 | 0.1340 | 0.1187 | -1.3089 | 0.8222 | 0.0131 | 0.0889 |
| rs34880121 | 0.4933 | 0.1146 | 0.1696 | -1.3646 | 0.8428 | -0.0043 | 0.0875 |
| rs237898 | 0.4366 | 0.0590 | 0.7329 | -1.2027 | 0.7743 | -0.0228 | 0.0864 |
| rs237899 | 0.4248 | 0.0516 | 0.8036 | -1.1267 | 0.7322 | -0.0304 | 0.0860 |
| rs237900 | 0.4266 | 0.0530 | 0.7884 | -1.1600 | 0.7553 | -0.0250 | 0.0863 |
| rs237902 | 0.3652 | 0.0747 | 0.5386 | -1.3974 | 0.8497 | -0.0039 | 0.0875 |
| rs4686302 | 0.2632 | 0.0236 | 0.9883 | -1.0063 | 0.6124 | -0.0530 | 0.0848 |
| rs115324487 | 0.0292 | 0.0408 | 0.5329 | -1.1687 | 0.7583 | 0.0173 | 0.0897 |
| rs2228485 | 0.4217 | 0.0238 | 0.9851 | -1.1187 | 0.7285 | -0.0313 | 0.0860 |
| rs112772544 | 0.0344 | 0.0490 | 0.5323 | -1.2027 | 0.7743 | 0.0008 | 0.0883 |
| rs237911 | 0.2718 | 0.0315 | 0.9515 | -1.1798 | 0.7611 | -0.0238 | 0.0864 |
| rs237913 | 0.2633 | 0.0850 | 0.4055 | -1.3453 | 0.8373 | 0.0088 | 0.0885 |
| rs7628496 | 0.0421 | 0.0716 | 0.3677 | -1.0967 | 0.7077 | 0.0377 | 0.0916 |
| rs114738868 | 0.0413 | 0.0729 | 0.3591 | -1.0805 | 0.7031 | 0.0394 | 0.0919 |
| rs237915 | 0.2707 | 0.0961 | 0.3125 | -1.1174 | 0.7246 | 0.0333 | 0.0907 |
| rs4564970 | 0.2249 | 0.0343 | 0.924 | -0.9754 | 0.5829 | -0.0573 | 0.0846 |
| rs62243375 | 0.1378 | 0.0688 | 0.5072 | -1.2955 | 0.8149 | 0.0121 | 0.0888 |
| rs35413809 | 0.2241 | 0.0366 | 0.9078 | -1.0187 | 0.6296 | -0.0492 | 0.0851 |
| rs73132856 | 0.2232 | 0.0356 | 0.9152 | -0.9891 | 0.5933 | -0.0534 | 0.0849 |
| rs2301261 | 0.2185 | 0.0246 | 0.9762 | -0.6966 | 0.4538 | -0.1421 | 0.0805 |
| rs968389 | 0.5011 | 0.0346 | 0.9307 | 0.1364 | 0.2026 | -0.5970 | 0.0583 |
| rs9853381 | 0.0294 | 0.0347 | 0.6005 | -1.0376 | 0.6651 | 0.0431 | 0.0924 |
| rs2301260 | 0.2122 | 0.0264 | 0.9684 | -0.4216 | 0.3453 | -0.2621 | 0.0746 |
| rs73132859 | 0.1116 | 0.0759 | 0.4325 | -1.3432 | 0.8350 | -0.0007 | 0.0878 |
| rs76884424 | 0.0241 | 0.0333 | 0.6221 | -1.1355 | 0.7359 | -0.0148 | 0.0873 |
| rs3806675 | 0.4011 | 0.0925 | 0.3596 | -0.8202 | 0.5044 | 0.0820 | 0.0954 |
| rs1465386 | 0.2082 | 0.0286 | 0.9569 | -0.6588 | 0.4145 | -0.1461 | 0.0801 |
| rs7610471 | 0.0473 | 0.0788 | 0.3399 | -0.9901 | 0.6031 | 0.0646 | 0.0945 |
| rs2268497 | 0.4866 | 0.0249 | 0.9788 | -0.3099 | 0.2648 | -0.3373 | 0.0713 |
| rs2268498 | 0.4736 | 0.0159 | 0.9991 | 0.6177 | 0.0872 | -1.0138 | 0.0419 |
| rs237916 | 0.5017 | 0.0552 | 0.7319 | -1.3847 | 0.8478 | -0.0008 | 0.0877 |
| rs237917 | 0.4951 | 0.0887 | 0.4004 | -1.3422 | 0.8339 | 0.0093 | 0.0885 |
| rs17049528 | 0.1856 | 0.0120 | 0.9969 | 0.8329 | 0.0658 | -1.1245 | 0.0376 |
| rs4643699 | 0.1500 | 0.0081 | 1 | 1.0450 | 0.0382 | -1.3273 | 0.0318 |
| rs1488466 | 0.2042 | 0.0188 | 0.9928 | -0.3263 | 0.3199 | -0.3172 | 0.0721 |
| rs1488467 | 0.1487 | 0.0081 | 1 | 1.0236 | 0.0503 | -1.3206 | 0.0321 |

**Table S2.** FDIST and BayeScan results for each tested locus of the AVPR1A gene. Left part shows expected heterozygosity (*H_E_*) and *F_ST_* values per loci for any significant (*P* (Simulation *F_ST_* < sample *F_ST_*) outlier locus under diversifying selection and balancing selection detected by FDIST. Right part of the table shows the α-factors and *F_ST_* values per locus detected by BayeScan. SNPs identified in BayeScan as under balancing (α_i_ < 0) and/or diversifying selection (α_i_ > 0) with the corresponding posterior estimate of *F_ST_* are shown as well as q-values; a Bayes Factor (BF) above 32 (i.e., log10(BF) > 1.5) correspond to a posterior probability of > 0.97 🡪 0.99 and thus indicates ‘very strong’ evidence for selection. Note: *F_ST_* - values differ due to different calculation algorithms.

| **Locus** | *H_E_* | ***F_ST_*** | ***P*** | **log10(BF)** | **q-val** | **α_i_** | ***F_ST_*** |
| --- | --- | --- | --- | --- | --- | --- | --- |
| rs75994003 | 0.0578 | 0.0892 | 0.4916 | -1.1466 | 0.6512 | -0.0208 | 0.0726 |
| rs57177493 | 0.0630 | 0.0844 | 0.5141 | -1.2520 | 0.7402 | -0.0002 | 0.0737 |
| rs139019791 | 0.2059 | 0.0653 | 0.8477 | -0.1498 | 0.2487 | -0.3832 | 0.0566 |
| rs140690814 | 0.2087 | 0.0284 | 0.9943 | 1.0787 | 0.0508 | -1.1405 | 0.0293 |
| rs61560121 | 0.2388 | 0.0291 | 0.9935 | 0.2236 | 0.1553 | -0.7159 | 0.0447 |
| rs12830859 | 0.2631 | 0.0143 | 1 | 1.1382 | 0.0455 | -1.2413 | 0.0271 |
| rs7966610 | 0.3140 | 0.0337 | 0.9926 | -0.7927 | 0.4147 | -0.1117 | 0.0686 |
| rs182223193 | 0.2700 | 0.0160 | 0.9947 | 0.2776 | 0.0929 | -0.7640 | 0.0430 |
| rs186484310 | 0.2448 | 0.0241 | 0.9707 | -1.0560 | 0.5289 | -0.0444 | 0.0713 |
| rs61353632 | 0.2185 | 0.1169 | 0.478 | -1.3359 | 0.7824 | 0.0006 | 0.0736 |
| rs7980289 | 0.2854 | 0.2496 | 0.0271 | -1.2917 | 0.7681 | 0.0154 | 0.0746 |
| rs1580704 | 0.1274 | 0.0226 | 0.9965 | -1.1600 | 0.6795 | -0.0248 | 0.0723 |
| rs1587098 | 0.1412 | 0.0111 | 1 | 0.2131 | 0.1957 | -0.6964 | 0.0452 |
| rs1580705 | 0.1311 | 0.0176 | 0.9992 | -0.4628 | 0.3333 | -0.2264 | 0.0635 |
| rs11174805 | 0.1412 | 0.0111 | 1 | 0.2240 | 0.1280 | -0.6969 | 0.0451 |
| rs11174806 | 0.1412 | 0.0111 | 1 | 0.2212 | 0.1773 | -0.6892 | 0.0453 |
| rs74097646 | 0.0273 | 0.0610 | 0.549 | -1.0787 | 0.5580 | 0.0052 | 0.0750 |
| rs56657448 | 0.2055 | 0.1027 | 0.5546 | -1.3779 | 0.8039 | -0.0054 | 0.0732 |
| rs56218817 | 0.2055 | 0.1027 | 0.5546 | -1.3859 | 0.8119 | -0.0036 | 0.0733 |
| rs61936378 | 0.3880 | 0.1953 | 0.0985 | -1.4353 | 0.8243 | 0.0033 | 0.0737 |
| rs142404469 | 0.0382 | 0.0721 | 0.5091 | -1.1362 | 0.6255 | 0.0080 | 0.0748 |
| rs9805119 | 0.2467 | 0.1741 | 0.1778 | -1.3288 | 0.7797 | 0.0084 | 0.0741 |
| rs962862 | 0.0909 | 0.0513 | 0.8418 | -0.8526 | 0.4587 | -0.0847 | 0.0695 |
| rs11174807 | 0.1431 | 0.0124 | 1 | 0.0089 | 0.2207 | -0.5322 | 0.0513 |
| rs1812476 | 0.2467 | 0.1741 | 0.1778 | -1.3679 | 0.7973 | 0.0066 | 0.0739 |
| rs1812477 | 0.3901 | 0.1944 | 0.1008 | -1.4642 | 0.8260 | 0.0026 | 0.0736 |
| rs144494446 | 0.0606 | 0.0915 | 0.4748 | -1.1850 | 0.6975 | -0.0163 | 0.0729 |
| rs112383228 | 0.0570 | 0.0875 | 0.5008 | -1.1680 | 0.6917 | -0.0216 | 0.0725 |
| rs7954346 | 0.2101 | 0.1061 | 0.5312 | -1.3613 | 0.7877 | -0.0015 | 0.0734 |
| rs113357563 | 0.0570 | 0.0875 | 0.5008 | -1.1501 | 0.6660 | -0.0219 | 0.0726 |
| rs7967970 | 0.0514 | 0.0807 | 0.539 | -1.1926 | 0.7030 | 0.0021 | 0.0741 |
| rs74097647 | 0.0475 | 0.0796 | 0.47 | -1.1396 | 0.6346 | 0.0211 | 0.0758 |
| rs7967990 | 0.2123 | 0.1106 | 0.5021 | -1.4010 | 0.8174 | -0.0022 | 0.0734 |
| rs74097649 | 0.0273 | 0.0610 | 0.549 | -1.1141 | 0.5835 | 0.0018 | 0.0745 |
| rs74097651 | 0.0475 | 0.0796 | 0.47 | -1.1501 | 0.6660 | 0.0222 | 0.0759 |
| rs1774 | 0.1603 | 0.1151 | 0.465 | -1.2661 | 0.7550 | 0.0145 | 0.0746 |
| rs11174808 | 0.0657 | 0.0969 | 0.4459 | -1.2778 | 0.7584 | 0.0016 | 0.0738 |
| rs10047514 | 0.4667 | 0.0847 | 0.7918 | -1.3701 | 0.7996 | -0.0005 | 0.0735 |
| rs11829406 | 0.1717 | 0.1792 | 0.1668 | -1.2452 | 0.7319 | 0.0198 | 0.0751 |
| rs10877967 | 0.1189 | 0.0699 | 0.7345 | -1.3278 | 0.7769 | -0.0017 | 0.0735 |
| rs11829452 | 0.1690 | 0.1769 | 0.1738 | -1.2138 | 0.7133 | 0.0197 | 0.0751 |
| rs10747983 | 0.3732 | 0.1520 | 0.2778 | -1.3485 | 0.7851 | -0.0026 | 0.0734 |
| rs10784339 | 0.3726 | 0.1505 | 0.2865 | -1.3813 | 0.8060 | -0.0035 | 0.0733 |
| rs59829368 | 0.1169 | 0.0868 | 0.6035 | -1.2879 | 0.7649 | 0.0004 | 0.0736 |
| rs118156056 | 0.0416 | 0.0140 | 0.993 | -1.2546 | 0.7441 | 0.0044 | 0.0740 |
| rs11174810 | 0.1704 | 0.1312 | 0.3714 | -1.3824 | 0.8080 | 0.0030 | 0.0737 |
| rs151155601 | 0.0224 | 0.0238 | 0.7978 | -1.1665 | 0.6857 | -0.0011 | 0.0739 |
| rs117072097 | 0.0272 | 0.0041 | 0.9987 | -1.1465 | 0.6512 | -0.0256 | 0.0724 |
| rs11174811 | 0.1467 | 0.0047 | 1 | 1.2742 | 0.0336 | -1.3077 | 0.0255 |
| rs3803107 | 0.2930 | 0.0655 | 0.8895 | -0.9005 | 0.4959 | -0.0686 | 0.0702 |
| rs10219543 | 0.4655 | 0.0630 | 0.931 | -1.3257 | 0.7741 | -0.0005 | 0.0735 |
| rs74097652 | 0.1176 | 0.0876 | 0.5974 | -1.3187 | 0.7711 | 0.0024 | 0.0738 |
| rs34310119 | 0.0738 | 0.0577 | 0.7544 | -0.9446 | 0.5127 | 0.0690 | 0.0797 |
| rs34462214 | 0.1549 | 0.0048 | 1 | 1.5306 | 0.0252 | -1.3346 | 0.0248 |
| rs74889535 | 0.0389 | 0.0252 | 0.9682 | -1.1233 | 0.6160 | 0.0320 | 0.0764 |
| rs73327840 | 0.0332 | 0.0503 | 0.6298 | -1.1550 | 0.6729 | -0.0061 | 0.0738 |
| rs73327843 | 0.0866 | 0.1269 | 0.3612 | -1.2367 | 0.7230 | 0.0158 | 0.0750 |
| rs10877968 | 0.3153 | 0.0581 | 0.9493 | -0.8858 | 0.4781 | -0.0754 | 0.0699 |
| rs10784341 | 0.4505 | 0.0236 | 0.9956 | -0.8502 | 0.4378 | -0.0907 | 0.0694 |
| 12:63544047 | 0.0065 | 0.0367 | 0.1404 | -0.7182 | 0.3630 | 0.1817 | 0.0939 |
| rs1042615 | 0.4687 | 0.0788 | 0.8377 | -1.3779 | 0.8039 | -0.0010 | 0.0734 |
| rs2228153 | 0.0874 | 0.1289 | 0.3524 | -1.1972 | 0.7082 | 0.0193 | 0.0752 |
| rs2228154 | 0.0874 | 0.1289 | 0.3524 | -1.2512 | 0.7361 | 0.0160 | 0.0749 |
| rs3021531 | 0.0874 | 0.1289 | 0.3524 | -1.2435 | 0.7275 | 0.0140 | 0.0747 |
| rs3741865 | 0.0650 | 0.0957 | 0.4521 | -1.2852 | 0.7617 | 0.0011 | 0.0738 |
| rs3021530 | 0.0884 | 0.1319 | 0.3396 | -1.2599 | 0.7478 | 0.0176 | 0.0751 |
| rs3021529 | 0.1567 | 0.0057 | 0.9999 | 1.2035 | 0.0399 | -1.2604 | 0.0266 |
| rs3021528 | 0.0661 | 0.0932 | 0.4655 | -1.2643 | 0.7515 | 0.0006 | 0.0737 |
| rs74097656 | 0.0350 | 0.0417 | 0.7101 | -1.1147 | 0.5950 | -0.0158 | 0.0732 |
| rs145866023 | 0.0439 | 0.0765 | 0.4859 | -1.1044 | 0.5712 | 0.0326 | 0.0766 |
| rs3087488 | 0.1821 | 0.0519 | 0.9173 | -0.2960 | 0.2784 | -0.3002 | 0.0602 |
| rs113697298 | 0.0246 | 0.0124 | 0.9724 | -0.3503 | 0.3059 | -0.4149 | 0.0587 |
| rs10877969 | 0.3660 | 0.1935 | 0.1026 | -1.3998 | 0.8156 | 0.0046 | 0.0738 |
| rs3759292 | 0.1552 | 0.2003 | 0.1136 | -0.7408 | 0.3899 | 0.1121 | 0.0832 |
| rs11174816 | 0.1812 | 0.1539 | 0.2504 | -1.3657 | 0.7950 | 0.0045 | 0.0738 |
| rs78784585 | 0.0135 | 0.0131 | 0.8188 | -1.1213 | 0.6058 | -0.0096 | 0.0736 |
| rs7308855 | 0.1114 | 0.0020 | 1 | 2.1158 | 0.0076 | -1.6966 | 0.0178 |
| rs7294536 | 0.3660 | 0.1935 | 0.1026 | -1.3847 | 0.8100 | 0.0046 | 0.0738 |
| rs11174817 | 0.4729 | 0.0717 | 0.891 | -1.3646 | 0.7926 | 0.0027 | 0.0737 |
| rs7298346 | 0.3617 | 0.1815 | 0.1404 | -1.4057 | 0.8192 | 0.0019 | 0.0736 |
| rs2738250 | 0.0823 | 0.0415 | 0.9179 | -1.0654 | 0.5440 | 0.0444 | 0.0773 |
| rs60766036 | 0.3647 | 0.1904 | 0.1114 | -1.4277 | 0.8226 | 0.0034 | 0.0737 |
| rs10877970 | 0.3727 | 0.2152 | 0.0551 | -1.3916 | 0.8138 | 0.0069 | 0.0740 |
| rs11174818 | 0.0642 | 0.1007 | 0.4265 | -1.2235 | 0.7182 | 0.0128 | 0.0746 |
| rs11174819 | 0.3631 | 0.1860 | 0.1251 | -1.4178 | 0.8209 | 0.0049 | 0.0738 |
| rs113481894 | 0.1868 | 0.0355 | 0.9866 | -1.3635 | 0.7902 | 0.0031 | 0.0738 |

## **Table S3**. Population collection of 1092 samples of 14 human populations of 1000 Genomes

## Project phase 1 integrated release version 3.

| **Population Code** | **Population Description** | **Sample Size** |
| --- | --- | --- |
| CHB | Han Chinese in Bejing, China | 97 |
| CHS | Southern Han Chinese | 100 |
| JPT | Japanese in Tokyo, Japan | 89 |
| CEU | Utah Residents (CEPH) with Northern and Western European ancestry | 85 |
| TSI | Toscani in Italia | 98 |
| GBR | British in England and Scotland | 89 |
| IBS | Iberian population in Spain | 14 |
| FIN | Finnish in Finland | 93 |
| YRI | Yoruba in Ibadan, Nigeria | 88 |
| LWK | Luhya in Webuye, Kenya | 97 |
| ASW | Americans of African Ancestry in SW USA | 61 |
| MXL | Mexican ancestry in Los Angeles, California | 66 |
| CLM | Colombians from Medellin, Colombia | 60 |
| PUR | Puerto Ricans from Puerto Rico | 55 |

**Table S4.** Results of the Lewontin and Krakauer test extended for the SNPs on the OXTR gene. Shown are the SNP locus, the estimated heterozygosity (Ht), Lewontion and Krackauer extended test statistics (F.LK) and *P* values for F.LK (F.LK.p.val).

| **Locus** | **Ht** | **F.LK** | **F.LK.p.val** |
| --- | --- | --- | --- |
| rs59746083 | 0.320 | 4.376 | 0.112 |
| rs56898713 | 0.306 | 4.191 | 0.123 |
| rs35014760 | 0.209 | 0.645 | 0.724 |
| rs2324728 | 0.422 | 3.965 | 0.138 |
| rs4493422 | 0.407 | 3.108 | 0.211 |
| rs9872310 | 0.282 | 1.367 | 0.505 |
| rs9872425 | 0.165 | 0.689 | 0.709 |
| rs11131147 | 0.143 | 1.010 | 0.604 |
| rs237884 | 0.424 | 3.316 | 0.190 |
| rs6770632 | 0.423 | 3.988 | 0.136 |
| rs1042778 | 0.467 | 0.142 | 0.932 |
| rs36047964 | 0.138 | 3.921 | 0.141 |
| rs2139184 | 0.157 | 5.057 | 0.080 |
| rs237885 | 0.499 | 0.000 | 1.000 |
| rs237886 | 0.495 | 0.112 | 0.945 |
| rs11706648 | 0.419 | 1.989 | 0.370 |
| rs11718289 | 0.447 | 3.065 | 0.216 |
| rs237887 | 0.468 | 1.311 | 0.519 |
| rs2268490 | 0.333 | 1.990 | 0.370 |
| **rs237888** | 0.267 | 8.993 | 0.011 |
| rs918316 | 0.204 | 2.717 | 0.257 |
| rs9840864 | 0.446 | 2.084 | 0.353 |
| rs4686301 | 0.407 | 2.236 | 0.327 |
| rs2268491 | 0.322 | 2.499 | 0.287 |
| rs2268492 | 0.371 | 2.231 | 0.328 |
| rs2300549 | 0.481 | 1.333 | 0.513 |
| rs2268493 | 0.374 | 2.046 | 0.360 |
| rs2268494 | 0.087 | 0.279 | 0.870 |
| rs2254298 | 0.313 | 2.355 | 0.308 |
| rs2254295 | 0.314 | 2.356 | 0.308 |
| rs60902022 | 0.492 | 1.033 | 0.597 |
| **rs57329700** | 0.272 | 6.818 | 0.033 |
| rs237889 | 0.380 | 3.907 | 0.142 |
| rs60345038 | 0.493 | 0.693 | 0.707 |
| rs13316193 | 0.489 | 1.089 | 0.580 |
| rs11131148 | 0.489 | 1.089 | 0.580 |
| rs62243369 | 0.305 | 2.336 | 0.311 |
| rs62243370 | 0.305 | 2.336 | 0.311 |
| rs11131149 | 0.484 | 0.680 | 0.712 |
| **rs59190448** | 0.262 | 13.521 | 0.001 |
| rs13319411 | 0.303 | 2.251 | 0.324 |
| rs237890 | 0.465 | 3.285 | 0.193 |
| rs12631502 | 0.267 | 2.804 | 0.246 |
| rs34992398 | 0.204 | 2.966 | 0.227 |
| rs237891 | 0.484 | 1.288 | 0.525 |
| rs53576 | 0.426 | 0.615 | 0.735 |
| rs237892 | 0.327 | 1.703 | 0.427 |
| rs35498753 | 0.283 | 1.080 | 0.583 |
| rs151463 | 0.460 | 1.456 | 0.483 |
| rs7652281 | 0.263 | 2.440 | 0.295 |
| rs237893 | 0.461 | 1.352 | 0.509 |
| rs11711703 | 0.280 | 2.290 | 0.318 |
| **rs73132848** | 0.196 | 6.697 | 0.035 |
| rs237894 | 0.290 | 3.079 | 0.215 |
| rs237895 | 0.433 | 1.825 | 0.402 |
| rs2268495 | 0.406 | 0.823 | 0.663 |
| rs78828026 | 0.132 | 2.140 | 0.343 |
| **rs61183828** | 0.198 | 6.757 | 0.034 |
| rs6767512 | 0.264 | 1.389 | 0.499 |
| rs2268496 | 0.411 | 0.934 | 0.627 |
| rs237897 | 0.427 | 1.650 | 0.438 |
| **rs79896191** | 0.196 | 6.566 | 0.038 |
| rs34880121 | 0.447 | 1.148 | 0.563 |
| rs237898 | 0.468 | 0.000 | 1.000 |
| rs237899 | 0.456 | 0.102 | 0.950 |
| rs237900 | 0.458 | 0.131 | 0.937 |
| rs237902 | 0.415 | 1.138 | 0.566 |
| rs4686302 | 0.260 | 2.374 | 0.305 |
| rs2228485 | 0.437 | 3.486 | 0.175 |
| rs237911 | 0.266 | 2.701 | 0.259 |
| rs237913 | 0.280 | 3.727 | 0.155 |
| rs237915 | 0.290 | 2.925 | 0.232 |
| rs4564970 | 0.252 | 1.202 | 0.548 |
| rs62243375 | 0.154 | 2.644 | 0.267 |
| rs35413809 | 0.253 | 1.223 | 0.543 |
| rs73132856 | 0.252 | 1.202 | 0.548 |
| rs2301261 | 0.239 | 0.969 | 0.616 |
| rs968389 | 0.497 | 0.280 | 0.869 |
| rs2301260 | 0.230 | 0.801 | 0.670 |
| rs73132859 | 0.154 | 3.343 | 0.188 |
| **rs3806675** | 0.359 | 6.698 | 0.035 |
| rs1465386 | 0.220 | 1.097 | 0.578 |
| rs2268497 | 0.490 | 1.887 | 0.389 |
| rs2268498 | 0.477 | 1.635 | 0.441 |
| rs237916 | 0.489 | 0.154 | 0.926 |
| rs237917 | 0.478 | 4.164 | 0.125 |
| rs17049528 | 0.184 | 0.564 | 0.754 |
| rs4643699 | 0.127 | 0.237 | 0.888 |
| rs1488466 | 0.214 | 1.060 | 0.589 |
| rs1488467 | 0.127 | 0.250 | 0.882 |

**Table S5**. Results of the Lewontin and Krakauer test extended for the SNPs on the AVPR1A gene. Shown are the SNP locus, the estimated heterozygosity (Ht), Lewontion and Krackauer extended test statistics (F.LK) and *P* values for F.LK (F.LK.p.val).

| **Locus** | **Ht** | **F.LK** | **F.LK.p.val** |
| --- | --- | --- | --- |
| rs10219543 | 0.455 | 2.599 | 0.273 |
| rs1042615 | 0.463 | 2.600 | 0.273 |
| rs10784341 | 0.445 | 0.850 | 0.654 |
| rs10877968 | 0.323 | 1.006 | 0.605 |
| rs10877969 | 0.377 | 2.584 | 0.275 |
| rs11174810 | 0.195 | 1.580 | 0.454 |
| rs11174811 | 0.136 | 0.622 | 0.733 |
| rs11174816 | 0.209 | 1.821 | 0.402 |
| rs11174817 | 0.463 | 2.532 | 0.282 |
| rs3021529 | 0.147 | 0.594 | 0.743 |
| rs3087488 | 0.182 | 0.473 | 0.789 |
| rs34462214 | 0.146 | 0.517 | 0.772 |
| **rs3759292** | **0.176** | **14.129** | **0.001** |
| rs3803107 | 0.306 | 1.006 | 0.605 |
| rs7294536 | 0.377 | 2.584 | 0.275 |
| rs7308855 | 0.101 | 0.477 | 0.788 |
| rs74097652 | 0.431 | 0.874 | 0.646 |
